# Glasgow Aneurysm Score: a predictor of long-term mortality following endovascular repair of abdominal aortic aneurysm?

**DOI:** 10.1186/s12872-021-02366-y

**Published:** 2021-11-19

**Authors:** Anıl Özen, Metin Yılmaz, Görkem Yiğit, İsa Civelek, Mehmet Ali Türkçü, Ferit Çetinkaya, Ertekin Utku Ünal, Hakkı Zafer İşcan

**Affiliations:** 1 Department of Cardiovascular Surgery, Ankara City Hospital, Ankara, Turkey; 2 Department of Cardiovascular Surgery, VM Medicalpark Hospital, Ankara, Turkey; 3 Department of Cardiovascular Surgery, Yozgat City Hospital, Yozgat, Turkey; 4 Department of Cardiovascular Surgery, Hitit University Faculty of Medicine, Çorum, Turkey

**Keywords:** Endovascular, Aortic aneurysm, Risk assessment, Analysis, Survival, Mortality

## Abstract

**Background:**

To evaluate the value of Glasgow Aneurysm Score (GAS) in predicting long-term mortality and survival in patients who have undergone endovascular aortic aneurysm repair (EVAR) for abdominal aortic aneurysm (AAA).

**Methods:**

A retrospective single-center study of 257 patients with non-ruptured AAA undergoing EVAR between January 2013 and 2021. GAS scores were compared between the survivors (group 1) and the long-term mortality (group 2) groups. Cox regression analysis was used to determine independent predictors of late mortality. Receiver operating characteristic curve (ROC) analysis was used to determine the optimum cut-off values of GAS values to determine the effect on late-mortality. Survival analysis was conducted using Kaplan-Meier.

**Results:**

The study included 257 patients with a mean age of 69.75 ± 7.75 (46–92), who underwent EVAR due to AAA. Average follow up period was 18.98 ± 22.84 months (0–88). Fourty-five (17.8%) mortalities occured during long-term follow-up. A past medical history of cancer resulted in a 2.5 fold increase in risk of long-term mortality (OR: 2.52, 95% CI 1.10–5.76; p = 0.029). GAS values were higher in group 2 compared to group 1 (81.02 ± 10.33 vs. 73.73 ± 10.46; p < 0.001). The area under the ROC curve for GAS was 0.682 and the GAS cut-off value was 77.5 (specificity 64%, p < 0.001). The mortality rates in patients with GAS < 77.5 and GAS > 77.5 were: 12.8% and 24.8% respectively (p = 0.014). Every 10 point increase in GAS resulted in approximately a 2 fold increase in risk of long-term mortality (OR: 1.8, 95% CI 1.3–2.5; p < 0.001). Five year survival rates in patients with GAS < 77.5 and > 77.5 were 75.7% and 61.7%, respectively (p = 0.013).

**Conclusions:**

The findings of our study suggests that an increase in GAS score may predict long-term mortality. In addition, the mortality rates in patients above the GAS cut-off value almost doubled compared to those below. Furthermore, the presence of a past history of cancer resulted in a 2.5 fold increase in long-term mortality risk. Addition of cancer to the GAS scoring system may be considered in future studies. Further studies are necessary to consolidate these findings.

## Introduction

Endovascular aortic aneurysm repair (EVAR) is a contemporary treatment modality used in abdominal aortic aneurysm (AAA) repair which is less invasive in nature when compared to open surgery [1]. EVAR possesses early mortality advantages and results in shorter duration of hospital stay. Nevertheless, reinterventions are more frequent following EVAR and lifelong follow-up is necessary [2].

Various scoring systems such as the Glasgow Aneurysm Score (GAS), Hardman Index (HI), Leiden score and Vanzetto score have been used to predict the outcomes after open surgical repair (OSR) of the AAA [3]. GAS is a practical and objective scoring system which was devised in the 1980–1990 s to predict mortality following elective open AAA repair [4]. Later on, it was also applied to patients undergoing EVAR [5, 6]. Eventhough it is a scoring sytem whose validity and reliability has previously been shown, studies on post-EVAR long-term follow-up outcomes predicted using the GAS, are scarce [5, 6].

This study aims to evaluate the value of GAS in predicting long-term mortality and survival in patients who have undergone EVAR for AAA. In addition, the aim was to define a cut-off value for GAS and investigate the patient outcomes according to this value.

## Materials and methods

### Study design

This study complied with the Declaration of Helsinki, and ethical approval was granted by the local institutional review board. Informed consent was obtained from all study participants.

This was a single centre, retrospective study of 257 patients diagnosed with non-ruptured infrarenal AAA, who had undergone EVAR between January 2013 and January 2021. Patients with AAAs with a diameter ≥ 5.5 cm, saccular morphology regardless of the diameter, or rapid aneurysm growth > 5mm over a 6 month period were eligible for EVAR. Exclusion criteria included: emergency admissions (mainly ruptured aneurysms), presence of thoracoabdominal aneurysms, dissecting aortic aneurysms, pseudoaneurysms, isolated iliac aneurysms, those who underwent open aneurysm repair procedures and hybrid revascularization. Technical details of stent deployment have been described previously [7]. The patients were followed up on an outpatient basis 1, 6, 12, 18 and 24 months post-EVAR, and annually thereafter. Those who did not come to the outpatient clinic for follow-up were contacted via telephone. According to the patient follow up, study participants were divided into two groups; the survivors (group 1) and the long-term mortality (group 2) patients (≥ 1 month post EVAR).

The primary outcome of this study was to determine the ability of GAS to predict long-term mortality. Secondary outcomes included determing independent risk predictors (such as age, sex, presence of cardiopulmonary comorbidities and malignancy) for long-term mortality. Patients’ demographic data included: age, sex and clinical features such as ejection fraction (EF) and other concomitant disorders such as previous cardiovascular, respiratory, renal, neurological disorders and past medical history of cancer. The clinical features of each patient was used to calculate the GAS score: the sum of age, +7 points for myocardial disease, +10 points for cerebrovascular disease, and +14 points for renal disease [4]. Myocardial disease included previously documented myocardial infarction or ongoing angina pectoris or both. Cerebrovascular disease included stroke and transient ischemic attack. Renal dysfunction was defined as a serum creatine level > 150 mmol/L or a urea > 20 mmol/ L or a history of acute or chronic renal failure or both [4].

Pre-procedural aneurysm diameter, based on computerised tomography (CT) evaluation, and GAS scores were compared between the groups. The groups were also compared with regards to operative and post-operative data (operation time, fluoroscopy time, volume of contrast medium used, length of intensive care unit (ICU) and hospital stay. Finally, the independent risk predictors for late-mortality were analysed. This was followed by survival analysis in the1st, 3rd and 5th years post-EVAR.

### Statistical analysis

All statistical analyses were performed using the SPSS statistical software (SPSS for Windows 15.0, Inc., Chicago, IL, USA). Continuous variables were tested for normal distribution using the Kolmogorov-Smirnov test. Normally distributed continuous variables were expressed as ‘mean values ± standard deviation (SD)’ or median values with the interquartile range if not normally distributed. Categorical variables were expressed as numbers and percentages. Demographic characteristics, perioperative variables and calculated values were compared using “independent samples *t*-test” or “Mann–Whitney-*U* test” for continuous variables and “chi-square test” or “Fisher’s exact test” for categorical variables. Correlations were assessed using Spearman’s correlation test. The odds ratios and 95% confidence intervals were estimated with Cox regression analysis to determine independent predictors of late mortality. All the variables that were found to be different at univariate analysis, were included in the Cox regression analysis Model (1) Model 1 also included the parameters of GAS rather than the GAS itself as this may act as a confounding factor. GAS values were used in the Cox regression analysis Model (2) GAS values as a predictor of long-term mortality were analysed using the reciever operating characteristic (ROC) analysis. When a significant cut-off value was observed, the sensitivity and specificity rates were presented. Kaplan-Meier analysis was conducted to demonstrate the survival. A p value < 0.05 was considered statistically significant.

## Results

### Patient demographics

Two hundred and fifty seven patients underwent EVAR due to AAA. A total of 49 mortalities occurred, 4 (1.5%) were in-hospital mortalities and were excluded from the study analysis. Fourty-five (17.8%) of the mortalities occured during the long-term follow-up. The average follow up period was 18.98 ± 22.84 months (0–88). Two hundred and eight patients (82.2%) were alive for the entirerity of the follow-up period.

Demographic characteristics of the patients are given in Table 1. There were no female patients in group 2 (0%, p < 0.029). The American Association of Anesthesiologists (ASA) Physical Status Classification System 1 and 2 were significantly higher in group 1 and ASA 3 and 4 were significantly higher in group 2 (p < 0.001). Moreover, incidence of COPD (p = 0.011), CAD (p < 0.020), CHF (p < 0.001) and history of cancer (p = 0.005) were all higher in group 2.

**Table 1:** Demographic characteristics of the patients

Characteristics	Group 1 (n = 208)	Group 2 (n = 4)	Total (n = 253)	P value
Age (years)	69.20 ± 7.79	72.33 ± 7.09	69.75 ± 7.75	0.031
EF (%)	53.19 ± 8.19	47.33 ± 12.33	52.16 ± 9.29	0.003
Female Sex	20 (9.6%)	0 (0.0%)	20 (7.9%)	0.029
Male Sex	188 (90.4%)	45 (100.0%)	233 (92.1%)	
ASA 1	15 (7.2%)	2 (4.4%)	17 (6.7%)	**< 0.001**
ASA 2	96 (46.2%)	13 (28.9%)	109 (43.1%)	
ASA 3	81 (38.9%)	16 (35.6%)	97 (38.3%)	
ASA 4	15 (7.2%)	14 (31.1%)	29 (11.5%)	
ASA 5	1 (0.5%)	0 (0.0%)	1 (0.4%)	
HT	150 (72.1%)	28 (62.2%)	178 (70.4%)	0.188
DM	58 (27.9%)	9 (20.0%)	67 (26.5%)	0.277
HL	58 (27.9%)	13 (28.9%)	71 (28.1%)	0.892
Smoking	101 (48.6%)	20 (44.4%)	121 (47.8%)	0.616
COPD	53 (25.5%)	20 (44.4%)	73 (28.9%)	0.011
CAD	81 (38.9%)	26 (57.8%)	107 (42.3%)	0.020
CHF	4 (1.9%)	8 (17.8%)	12 (4.7%)	**< 0.001**
TIA/CVS	13 (6.3%)	3 (6.7%)	16 (6.3%)	1.000
CRF	17 (8.1%)	10 (22.2%)	27 (10.6%)	0.360
PAD	15 (7.2%)	4 (8.9%)	19 (7.5%)	0.754
Cancer	7 (3.4%)	7 (15.6%)	14 (5.5%)	0.005
Symptomatic	61 (29.3%)	17 (37.8%)	78 (30.8%)	0.266

Statistically significant P values are given in bold

Values are mean ± SD or number (percentage in total). EF, Ejection fraction; HT, Hypertension; HL, Hyperlipidemia; DM, Diabetes mellitus; COPD, Chronic obstructive pulmonary disease; CAD, Coronary artery disease; CHF, Congestive heart failure; TIA/CVS, Transient ischemic attack/cerebrovascular stroke; CRF, Chronic renal failure; PAD, Peripheral artery disease; ASA, American Society of Anesthesiologists classification

### Comparison of GAS, aortic diameter and intra and postoperative data

Comparison of GAS, aortic diameter and intra and postoperative data between the groups are summarized in Table 2. GAS values were significantly higher in group 2 compared to group 1 (81.02 ± 10.33 vs. 73.73 ± 10.46; p < 0.001). There was no correlation between the GAS values and in-hospital mortality (p = 0.091). There was no significant difference in aortic diameters between the two groups (p = 0.134). ICU and hospital stays were longer in group 2 (p < 0.001 and p = 0.002, respectively). In addition, there was a weak correlation between GAS and ASA, and duration of ICU stay (r = 0.157; r = 0.593, respectively). A strong correlation was found between the GAS and the duration of hospital stay (r = 0.593).

**Table 2:** Comparison of GAS, aortic diameter and intra and postoperative data between the groups

Variables	Group 1 (n = 208)	Group 2 (n = 45)	Total (n = 253)	P value
GAS	73.73 ± 10.46	81.02 ± 10.33	75.05 ± 10.79	< **0.001**
Aneurysm diameter/mm	63.32 ± 12.71	66.33 ± 13.69	63.92 ± 12.94	0.134
Operation time/min	135.68 ± 49.37	135.00 ± 36.91	135.56 ± 47.22	0.767
Fluoroscopy time/min	17.08 ± 10.87	18.85 ± 10.66	17.43 ± 10.82	0.013
Amount of contrast medium /cc	63.59 ± 22.45	67.15 ± 23.18	64.28 ± 22.58	0.238
ICU stay/hour	6.26 ± 7.24	17.27 ± 38.72	8.25 ± 18.11	<**0.001**
Hospital stay/day	2.79 ± 2.94	4.00 ± 3.56	3.00 ± 3.08	**0.002**

Statistically significant P values are given in bold

Values are mean ± SD. GAS, Glasgow Aneurysm Score; ICU, Intensive care unit

### Cox Regression

Cox regression analysis of the groups in terms of GAS and GAS parameters, revealed that GAS and a history of cancer were independent risk predictors for long-term mortality. The Cox regression model (model 2) that included the GAS as a variable, revealed that a past medical history of cancer resulted in a 2.5 fold increase in risk for long-term mortality (OR: 2.52, 95% CI 1.10–5.76; p = 0.029). The same model also displayed that, every 1 point increase in GAS score resulted in a 1.06 fold increase in risk of late mortality (OR: 1.06, 95% CI 1.03–1.09; p < 0.001). Moreover, every 10 point increase in GAS score resulted in almost a 2 fold increase in risk of long-term mortality (OR: 1.8, 95% CI 1.3–2.5; p < 0.001). The findings of this analysis are summarized in Table 3. No correlation was found between GAS and endoleak. A low-grade positive correlation was found between GAS and ASA (r = 0.384, p < 0.001).

**Table 3:** Cox regression analysis for assesment of independent risk predictors for late-mortality

	Univariate	p value	Cox model 1	p value	Cox model 2	p value
Age	1.06 (1.01–1.11)	0.010	1.02 (0.92–1.14)	0.110		
Sex	–	0.998				
ASA	–	1.000				
COPD	2.34 (1.20–4.55)	0.012	1.38 (0.73–2.64)	0.326	1.67 (0.92–3.05)	0.094
CAD	2.15 (1.12–4.13)	0.022	0.96 (0.41–2.27)	0.928		
CHF	11.03 (3.16–38.50)	<0.001	3.04 (0.80–11.48)	0.102		
TIA/CVS	0.93 (0.26-3.42)	0.917				
Cancer	5.29 (1.76–15.95)	0.003	1.93 (0.73–5.12)	0.186	2.52 (1.10–5.76)	**0.029**
CRF	3.43 (1.44-8.17)	0.005	0.95 (0.21–4.27)	0.951		
GAS	1.07 (1.03–1.10)	< 0.001	1.03 (0.95–1.13)	0.480	1.06 (1.03–1.09)	< **0.001**
EF	0.94 (0.91–0.97)	< 0.001	0.98 (0.93–1.02)	0.339		
Fluoroscopy	1.01 (0.99–1.04)	0.350				

Statistically significant P values are given in bold

EF, Ejection fraction; COPD, Chronic obstructive pulmonary disease; CAD, Coronary artery disease; CHF, Congestive heart failure; TIA/CVS, Transient ischemic attack/cerebrovascular stroke; PAD, Peripheral artery disease; ASA, American Society of Anesthesiologists classification; CRF, Chronic renal failure

### ROC analysis

**Fig. 1:**
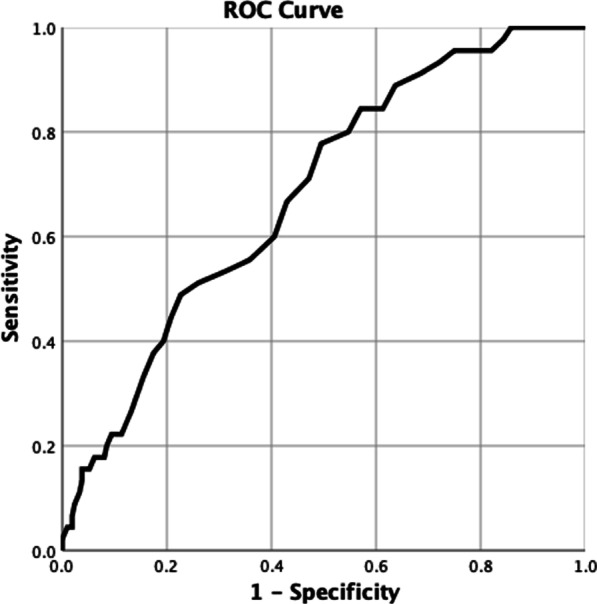
ROC-curve analysis showing the roc-curve formed at different threshold values

The area under the ROC curve for GAS was 0.682 and for a 56% sensitivity rate, the cut-off value for GAS was 77.5 with a specificity of 64% (p < 0.001). The findings of this analysis are given in Fig. 1. The number of patients with GAS values < 77.5 was 155, whilst it was 98 with GAS values > 77.5. The mortality rates in patients with GAS < 77.5 and GAS > 77.5 were: 12.8% (n = 20) and 24.8% (n = 25), respectively (p = 0.014). However, the mean hospital stay was not statistically significant in patients with GAS < 77.5 and >77.5 (2.83 ± 2.688 vs. 3.13 ± 3.526) (p:0.587).

Causes of death during follow-up were; cardiovascular in 23, aneursysm related in 12, cancer in 4, renal failure in 2, neurological in 2 and peripheral vascular disease in 2 patients.

### Survival analysis

**Fig. 2:**
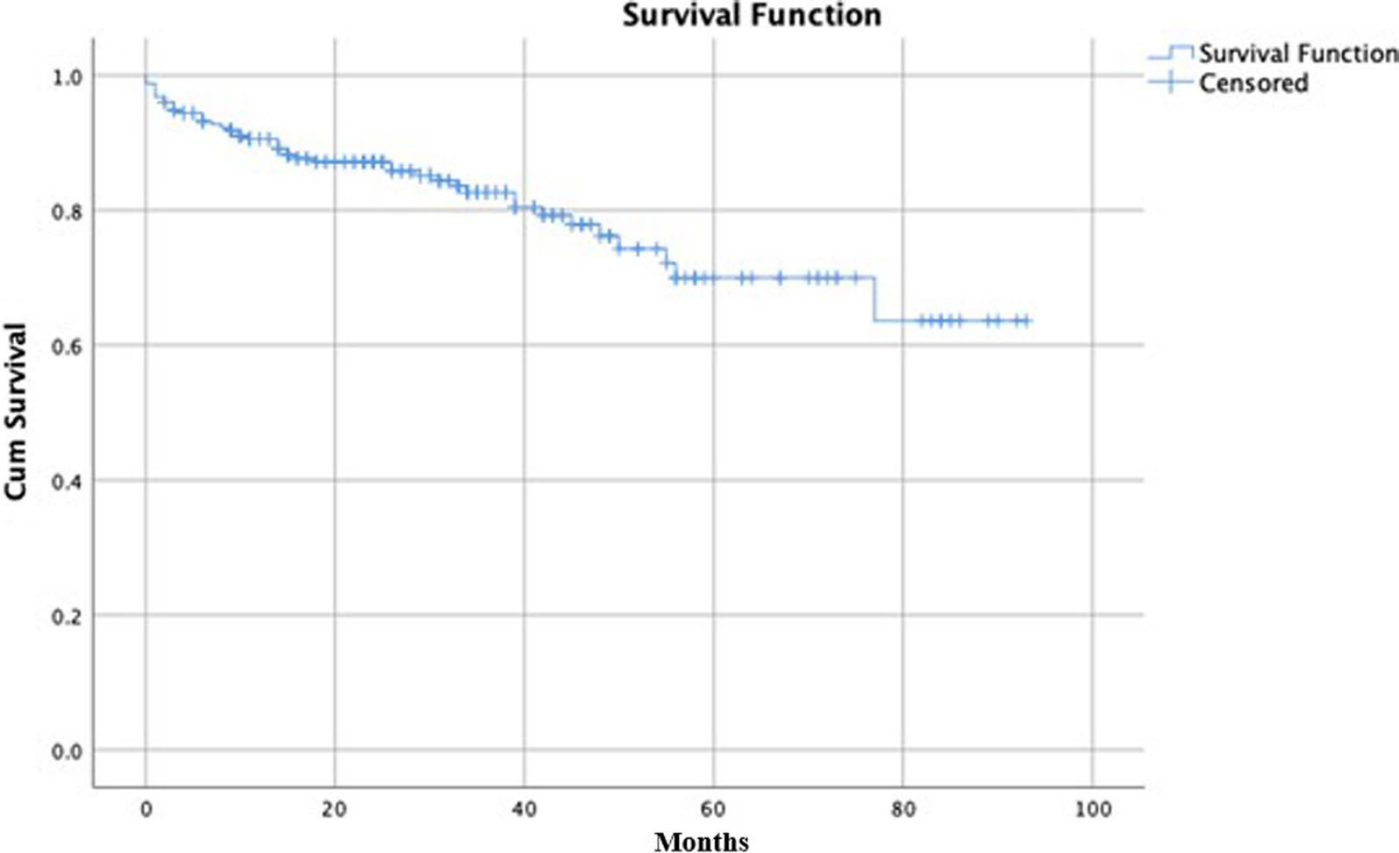
Survival analysis for the study population

**Fig. 3:**
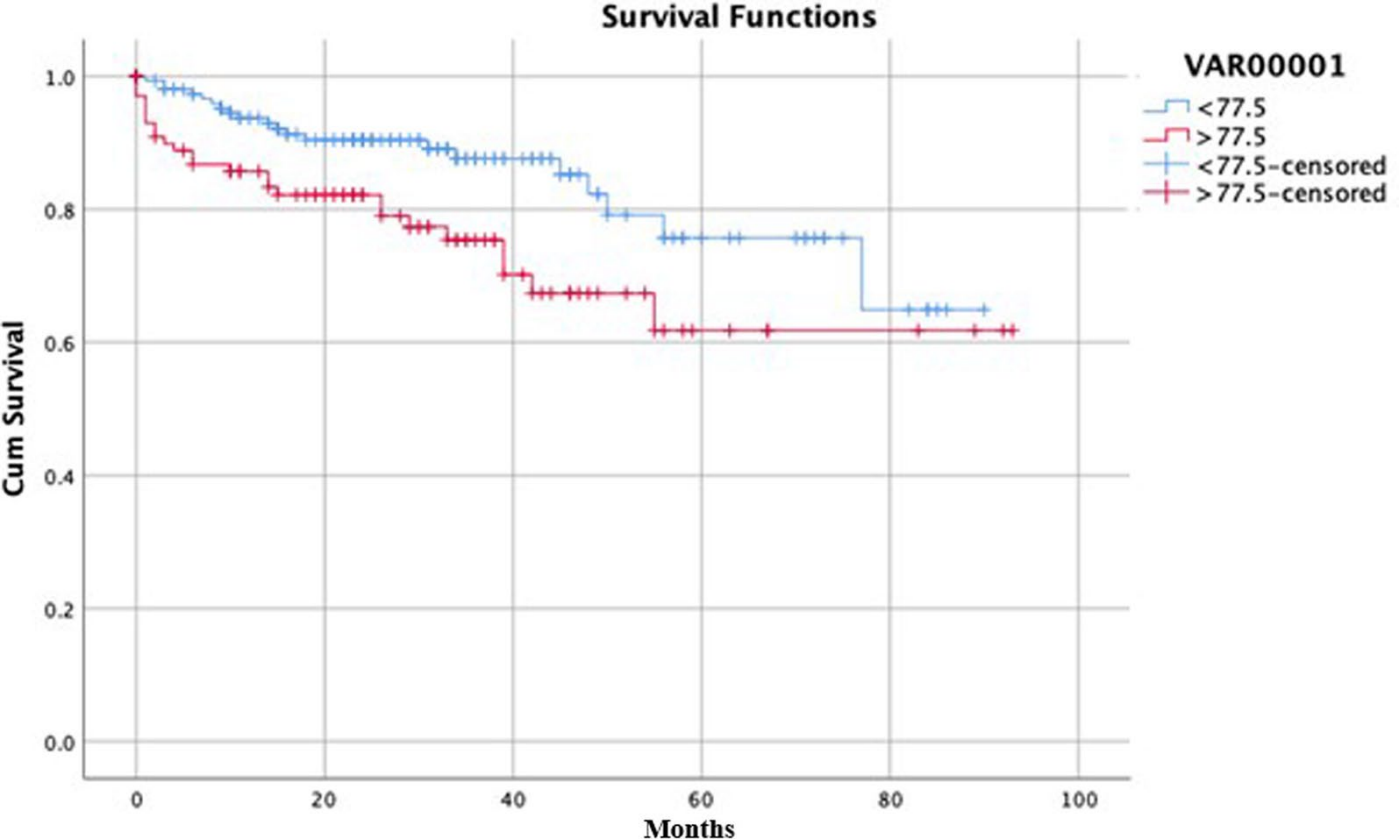
Kaplan-Meier analysis comparing survival rates in patients with GAS < 77.5 and > 77.5 (p:0.013)

The mean survival rate of the patients during the follow up period was 90.6% in the 1st year, 82.6% in the 3rd year and 70.0% in the 5th year (Fig. 2). Five year survival rates in patients with GAS < 77.5 and > 77.5 were 75.7% and 61.7%, respectively (p = 0.013) (Fig. 3).

## Discussion

In this study, we assessed the performance of GAS in predicting long-term outcome in elective AAA patients who had undergone EVAR. The long-term mortality rate in patients with GAS > 77.5, was almost twice that of the patients with GAS < 77.5. In addition, every 10 point increase in GAS score resulted in almost a two fold increase in risk of long-term mortality. Finally, the five year survival rate in patients with GAS > 77.5 was significantly less than in those with GAS scores < 77.5.

There are various risk scoring systems used in the prediction of mortality in patients undergoing repair for AAA. Despite the development of a few new scoring systems within the last decade, GAS still preserves its value in predicting outcomes following EVAR. Our results also show that GAS has predictive value during long-term follow-up in patients undergoing EVAR for AAA. A systematic review of 608 studies revealed that the most frequently used scoring systems for elective AAA repair were GAS, POSSUM and VBHOM. However, GAS was found to be the most used and validated scoring system for open repair [8]. Moreover, Nesi et al. [3] published an article in 2004, in which they compared four different scoring systems with GAS. They concluded that GAS was the most useful scoring system and the easiest one to apply in order to predict AAA surgical outcome. Previous studies conducted in our centre also support the use of GAS in patients undergoing elective and ruptured AAA surgery [9, 10].

Nevertheless, Visser et al. modified GAS by adding the type of procedure perfomed and evaluated predicted 30-day mortality in patients with ruptured AAAs treated with EVAR versus surgical AAA repair [11]. Similarly, Choke et al. developed an in-house risk prediction model, in order to predict perioperative mortality following elective AAA repair. They believed that the new models had better performance in predicting perioperative mortality for elective opensurgery and EVAR [2].

Most significantly, there are only a few studies in the literature regarding whether or not GAS predicts long-term survival following EVAR [5, 6]. The mean survival rates in our study were 90.6% for the 1st year, and 70.0% for the 5th year. Similarly, according to the EUROSTAR registry, the overall 1 and 5-year survival rates are 91.7% and 76.7%; this is one of the few studies with results regarding long-term follow-up after EVAR [5]. Moreover, in the current study, the five year survival rate in patients with GAS > 77.5 was significantly less than in those with GAS < 77.5. Likewise, the EUROSTAR registry revealed a 5-year overall survival rate of 65.2% in patients with GAS above the cut-off value of > 83.6 [5]. These results indicate that the survival rate decreases as the GAS values exceed the cut-off values. Furthermore, the EUROSTAR registry defined the 30-day mortality rate as 6.4% and 1.6% in patients with a score above and below the cut-off value respectively [5]. However, long-term mortality rates were not presented in their study. In the present study, the long term mortality rate in patients with GAS > 77.5, was almost twice that of the patients with GAS < 77.5. Besides, every 10 point increase in GAS score resulted in almost a 2 fold increase in risk of long-term mortality.

The DREAM trial is the only randomized study comparing AAA patients treated with open repair and EVAR. In this study, Baas et al. concluded that the GAS can be used to predict 30-day and 2-year mortality for both treatment modalities [6]. There was no correlation between GAS values and in-hospital mortality in both the DREAM trial and our study. This is most likely due to the low number of early mortalities. In addition, the DREAM trial supports the idea that GAS was superior in predicting 30-day and 2-year mortality in patients treated with EVAR compared to OR [6]. Their cut-off value for GAS for EVAR, during 2 year follow-up was also the same as ours at 77.5%.

According to the results of our study, there were no female long-term mortalities. In contrast, the study by Tümer et al. revealed no difference in all-cause mortality between the genders during the long-term follow-up period following EVAR [12]. We believe that the abscence of female patients in the long term mortality group of our study may have been due to the comparatively lower number of study participants compared to previous multi-center studies.

The American Association of Anestesiologists (ASA) Physical Status Classification System is one of the grading methods applied in order to categorize the patients’ preoperative status as well as to assess postoperative morbidity and mortality [13, 14]. Another finding of our study was the higher ratio of ASA 3 and 4 patients in the long-term mortality group. According to a study published in 2019, ICU and hospital stay were longer in ASA 3 and 4 patients. However, 1 year mortality and morbidity rates did not differ significantly between low and high risk ASA groups [15]. In contrast, our results showed that long-term mortality rates were higher in the high risk ASA 3 and 4 groups and in addition, the duration of ICU stay was weakly correlated with the ASA grades.

Despite a strong correlation found between the GAS and the duration of post-EVAR hospital stay, the mean post procedural hospital stay was not statistically significant in patients with GAS < 77.5 and >77.5. The most important reason for this was that ten of the patients who underwent EVAR with a GAS < 77.5, had an unexpectedly high hospital stay: four due to renal, 3 due to cardiac and 3 due to periperal arterial complications.

Another interesting finding of our study was that a medical history of cancer resulted in a 2.5 fold increase in risk for long-term mortality. A long-term outcome study of AAA patients, by Ahn et al. documented that mortality was significantly higher in patients with a history of malignancy [16]. The rate of cancer was four times higher in the long-term mortality group compared to the survivors group in the current study. However, the cause of death was primarily cardiovascular diseases followed by aneurysm related causes in the late-term mortality group. Cancer was the third common cause of mortality; out of 7 cancer patients in the long-term mortality group, 4 died. The presence of CHF was 9 times higher and CAD was 1.5 times higher in the long-term mortality group compared to the survivors group, which explains why cardiovascular etiologies were the leading cause of death in this study.

The results of our study support that GAS continues to be of value in the prediction of AAA outcome. The easy applicability of the GAS enables its use during long-term follow-up and may help in the education and counselling of patient peri-operatively regarding the long-term risks of EVAR.

## Limitations

The sensitivity and specificity rate for the cut-off value for GAS (56% and 64% respectively) may seem low and less predictive than expected. However, we believe that these values are reasonable for predicting the mortality risk in the long-term follow-up. Similar values have been reported in previous studies [5, 10, 17].

## Conclusion

The findings of our study suggest that an increase in GAS score may predict long-term mortality. Every 10 point increase in GAS score resulted in almost a 2 fold increase in risk of long-term mortality. Mortality rates in patients above the GAS cut off value almost doubled compared to those below. Furthermore, the current study revealed that the presence of a past history of cancer resulted in a 2.5 fold increase in long-term mortality risk. Indeed, combining the history of cancer with the GAS scoring system as part of a new prediction model may be considered. The outcomes of such a model can be analysed in future studies involving larger patient populations.

## Data Availability

The datasets used and/or analysed during the current study are available from the corresponding author on reasonable request.
